# Dementia CARES: A Training Series to Improve Caregiver Access to Resources, Education and Support

**DOI:** 10.1093/geroni/igaf122.605

**Published:** 2025-12-31

**Authors:** Sara Murphy, Jane Oderberg, Jennifer Severance, Janice Knebl

**Affiliations:** University of North Texas Health Science Center, Fort Worth, Texas, United States; University of North Texas Health Science Center, Fort Worth, Texas, United States; University of North Texas Health Science Center - Fort Worth, TX, Fort Worth, Texas, United States; University of North Texas Health Science Center, Fort Worth, Texas, United States

## Abstract

The entry point into dementia caregiving is a critical time in which a caregiver needs information, emotional and psychological support, and assistance in planning for their future role. The Dementia CARES series introduces dementia caregivers to strategies and topics to promote social support and the acquisition of new skills and knowledge. Session topics include 1) living with dementia; 2) behavioral changes; 3) strategies to form a local support network; 4) improving communication with the person living with dementia and their care team; 5) exploring resources, including evidence-based programs and clinical trials; and 6) practicing self-nurturing activities. Each session involves group discussion to learn about a dementia topic followed by sharing and problem-solving activities to practice new concepts, self-evaluation, and peer support to build caregiver confidence (self-efficacy) in carrying out tasks and addressing challenges. Designed by a licensed Master’s degree-level social worker at a geriatric primary care clinic, Dementia CARES is delivered as six one-hour virtual weekly group sessions for six to ten dementia caregivers. Four virtual series of the six-session CARES program were conducted in December 2018 to July 2019. Thirty-nine dementia caregivers were recruited by the primary care clinic, with twenty-nine (74%) participants completing at least four sessions. Preliminary data from post-intervention survey responses (n = 29) show 96.5% would strongly recommend the training to a friend or relative. Participants valued the discussion with other caregivers, learning strategies and suggestions, and sharing their experiences. Findings underscore the value and feasibility of offering structured group education and support in primary care settings.

